# Resveratrol Hinders Postovulatory Aging by Modulating Oxidative Stress in Porcine Oocytes

**DOI:** 10.3390/molecules26216346

**Published:** 2021-10-20

**Authors:** Benazir Abbasi, Yan Dong, Rong Rui

**Affiliations:** College of Veterinary Medicine, Nanjing Agricultural University, Nanjing 210095, China; benazirabbassi@gmail.com (B.A.); 2016107099@njau.edu.cn (Y.D.)

**Keywords:** resveratrol, postovulatory aging, oocyte quality, oxidative stress, reactive oxygen species

## Abstract

Postovulatory aging of the mammalian oocytes causes deterioration of oocytes through several factors including oxidative stress. Keeping that in mind, we aimed to investigate the potential of a well-known antioxidant, resveratrol (RV), to evaluate the adverse effects of postovulatory aging in porcine oocytes. After in vitro maturation (IVM), a group of (25–30) oocytes (in three replicates) were exposed to 0, 1, 2, and 4 μmol/L of RV, respectively. The results revealed that the first polar body (PB1) extrusion rate of the oocytes significantly increased when the RV concentration reached up to 2 μmol/L (*p* < 0.05). Considering optimum RV concentration of 2 μmol/L, the potential of RV was evaluated in oocytes aged for 24 and 48 h. We used fluorescence microscopy to detect the relative level of reactive oxygen species (ROS), while GHS contents were measured through the enzymatic method. Our results revealed that aged groups (24 h and 48 h) treated with RV (2 μmol/L) showed higher (*p* < 0.05) ROS fluorescence intensity than the control group, but lower (*p* < 0.05) than untreated aged groups. The GSH content in untreated aged groups (24 h and 48 h) was lower (*p* < 0.05) than RV-treated groups, but both groups showed higher levels than the control. Similarly, the relative expression of the genes involved in antioxidant activity (*CAT*, *GPXGSH-Px*, and *SOD1)* in RV-treated groups was lower (*p* < 0.05) as compared to the control group but higher than that of untreated aged groups. Moreover, the relative mRNA expression of caspase-3 and Bax in RV-treated groups was higher (*p* < 0.05) than the control group but lower than untreated groups. Furthermore, the expression of *Bcl-2* in the RV-treated group was significantly lower than control but higher than untreated aged groups. Taken together, our findings revealed that the RV can increase the expression of antioxidant genes by decreasing the level of ROS, and its potent antiapoptotic effects resisted against the decline in mitochondrial membrane potential in aged oocytes.

## 1. Introduction

The postovulation oocyte quality is the main factor that affects the efficiency of assisted reproductive technologies (ART) such as somatic cell nuclear transfer (SCNT), intracytoplasmic sperm injection (ICSI), and in vitro fertilization (IVF) [1,2]. The quality of an oocyte is mainly affected by structural and functional changes induced during aging including chromosome and spindle anomalies [3], cortical granule exocytosis [4], lower fertilization rates [5], zona pellucida (ZP) hardening [6], and abnormal or retarded development of embryos/fetuses [7,8]. The exact molecular mechanism underlying the reduced competence of an oocyte due to postovulatory aging is not fully understood. However, there are some major factors that mediate time dependent reduction in oocyte competence such as oxidative stress [9], chromosomal abnormalities [10], and modification of poly (A) tails (Deadenylation) of genes responsible for maternal effects [11] and epigenetic alteration [12,13]. Therefore, it is imperative to better understand the various mechanisms responsible for the postovulatory aging process to devise effective strategies to delay oocyte aging process and increase the time required for performing normal reproductive functions [9,14]. Oxidative stress is strongly associated with a deterioration in oocyte quality because it significantly reduces the glutathione (GSH) contents and assists in the accumulation of reactive oxygen species (ROS). The ROS such as superoxide anions (O^−2^), hydroxyl radicals (OH^−^), and hydrogen peroxide (H_2_O_2_) are released during normal metabolic (intermediate steps of oxygen reduction) processes [15,16]. The mitochondrion is the major cell organelle responsible for ROS production [17,18]. A dynamic balance is required between ROS production and antioxidant enzymes to ensure proper cellular homeostasis including cell proliferation, host defense, signal transduction, and gene expression [19]. The antioxidant defense system disrupted through the overproduction of ROS, which, in turn, causes oxidative stress. Excessive load of ROS results in proapoptotic signaling, subsequently leading to the activation of cell apoptosis [20]. Postovulatory aging is associated with excessive accumulation of ROS leading to oxidative stress, which predisposes aged oocytes to the apoptotic process [9,21]. The mitochondria as the major “energy generators” have a significant role in regulating proper function and survival of oocytes. However, being a prime source of ROS production, mitochondria are susceptible to ROS-induced damage [22], which results in the decreased ATP synthesis, altered mitochondrial membrane potential, oxidative stress, and early onset of apoptosis [23,24]. The excessive accumulation of ROS can affect the permeability of mitochondrial membranes to open MPTP (1-methyl-4-phenyl-1,2,3,6-tetrahydropyridine) and promote the flow of calcium ions [25], which subsequently induces the release of cytochrome C and caspase 3 activation leading to the apoptosis [26,27]. The apoptotic activation is mainly induced by the glutathione efflux [28], which leads to several morphological changes including cell shrinkage, progressive DNA, and cell membrane damage, ultimately leading to the cell death [29]. Therefore, one of the major challenges in reproductive embryology is to prevent oocytes’ degeneration to maintain their developmental competences [30]. To avoid oxidative damage by maintaining a robust antioxidant defense system in the oocyte, supplementation of exogenous antioxidants can be used as the most effective strategy. 

Resveratrol (3,5,4′-trihydroxyl-Trans-stilbene) (RV) is a stilbenoid, a type of natural polyphenolic compound with excellent antioxidant and free radical scavenging capacity. It is associated with reduced ROS accumulation, scavenges superoxide radicals, inhibits lipid peroxidation, and regulates the expression of antioxidant cofactors and enzymes [31]. Natural antioxidants that are effective may provide novel and safe interventional strategies to delay or prevent oocyte aging and related diseases. Porcine oocytes can be used as an ideal model in the field of reproductive biology, as they have much similar developmental and physiological properties as with human oocytes [32]. Therefore, this study was conducted to evaluate the effect of RV on pig oocytes during aging and to provide mechanistic insights regarding its potential of protecting oocytes against ROS attack. Reducing oxidative stress in the oocytes is an important way to slow down oocyte aging. Still, there is lack of data regarding the rescue of oocytes during aging. The underlying mechanisms of oxidative stress during oocyte aging as well as the protective mechanisms of the natural antioxidants in antiaging are thoroughly explored in the present study.

## 2. Results

### 2.1. RV Treatment Reverses Aging-Induced Impairment in Aged Porcine Oocytes 

**Figure 1:**
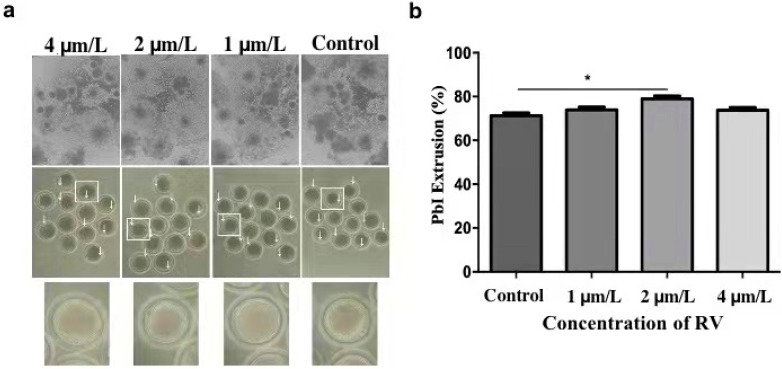
RV treatment reverses aging-induced impairment in aged porcine oocytes. (**a**) The representative images showing degrees of cumulus spread and first polar body extrusion rate, as indicated by white arrows. (**b**) The graph showing PB1 extrusion percentage rate at various concentrations (0, 1, 2, and 4 µmol/L RV). Significant difference (* *p* < 0.05).

For determining the optimal concentration of RV, which can delay oocyte age dependent impairments, the cumulus cells incubated for 44 h were removed with 0.1% (*w*/*v*) hyaluronidase using 37 °C for 3 min. The oocytes with even granular cytoplasm and a first polar body were selected for the subsequent experiments. During in vitro maturation, a total of 25–30 oocytes (in three replicates) for each group were cultured with 0, 1, 2, and 4 µmol/L RV, respectively. After maturation, the proliferation rate of cumulus oocyte complexes (COCs) was observed under a stereomicroscope. As shown in Figure 1a, most of the COCs showed fully expanded peripheral layers of cumulus in 1 and 2 µmol/L RV-treated groups, whereas the cumulus proliferation of COCs was significantly decreased in the control and 4 µmol/L of RV-treated group. In addition, a large proportion of the RV-treated oocytes failed to extrude the PB1 in a dose-dependent manner. As shown in Figure 1b, percentage of the PB1 extrusion rate was significantly higher in the 2 µmol/L RV-treated group (78.99 ± 1.07) as compared to the control group (71.26 ± 1.02%). However, there was no significant difference observed for these parameters in 1 and 4 µmol/L RV-treated groups (73.95 ± 1.05% and 73.76 ± 1.02, respectively). 

### 2.2. RV Suppresses the Increasing Perivitaline Space (PVS) in Aged Porcine Oocytes

**Figure 2:**
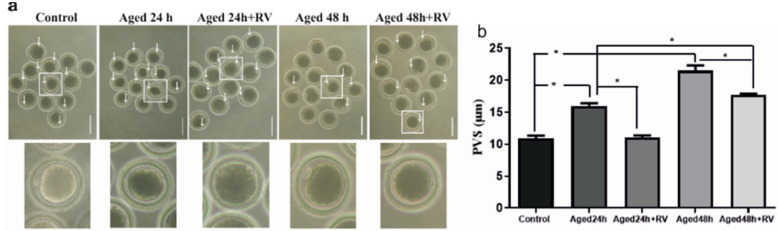
RV suppresses the increasing perivitaline space (PVS) in aged porcine oocytes (**a**) The representative images of perivitaline space in aged porcine oocytes with visible space indicated by white arrows under the microscope, scale bar = 180 µm. (**b**) The graph indicates the percent increase in perivitaline space after the oocytes treated with 2 µmol/L RV. Significant difference (* *p* < 0.05).

As shown in Figure 2a, arrows indicate the significantly increased perivitaline space in oocytes of 24 h and 48 h aged groups (15.98 ± 0.60 and 21.51 ± 1.16, respectively) as compared to the control group (10.94 ± 0.53). Moreover, the treatment with 2 µmol/L of RV prolonged oocyte culture (24 h and 48 h) can significantly suppress the perivitaline space when compared with untreated (24 h and 48 h) aged groups.

### 2.3. RV Reduces the Apoptosis Extent in Aged Porcine Oocytes

**Figure 3:**
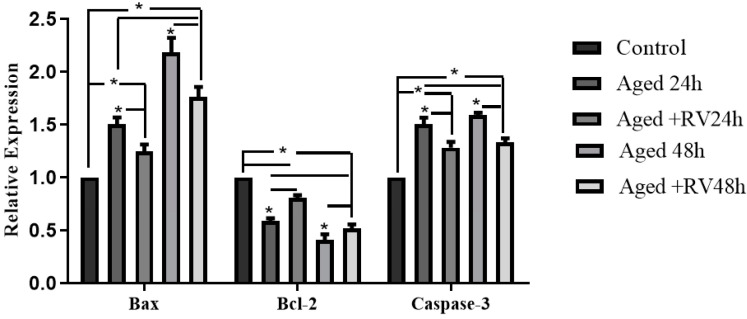
The relative mRNA expression of apoptosis related (Caspase-3, Bcl-2, and Bax) genes in aged porcine oocytes at different time periods. Significant difference (* *p* < 0.05).

The apoptosis includes a series of cellular apoptotic events that occur during oocyte aging during in vitro maturation [33]. As shown in Figure 3 the mRNA expression of apoptosis related genes (Caspase-3, Bax, and Bcl-2) were analyzed through qRT-PCR to determine the cellular activities during the RV treatment. The results showed that the mRNA levels of Caspase-3 and Bax treated with 2 µmol/L RV were significantly higher than those in the control group but significantly lower than those in the 24 h and 48 h aged groups, while the expression of Bcl-2 was significantly lower. Moreover, the expression of Bcl-2 group oocytes treated with 2 µmol/L RV was significantly lower than that in control but significantly higher than that in the untreated aged groups. 

### 2.4. RV Alleviates the Oxidative Stress in Aged Porcine Oocytes

**Figure 4:**
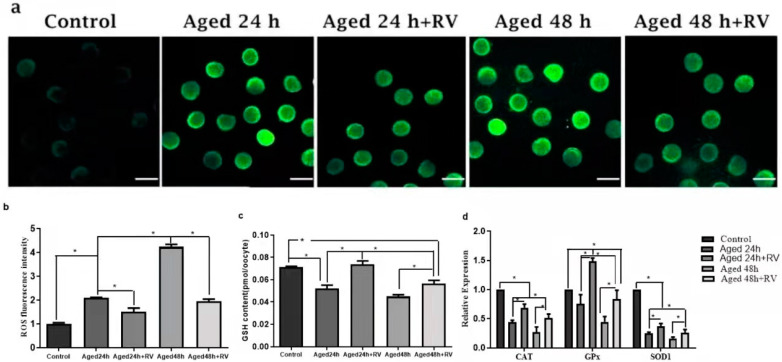
RV alleviates the oxidative stress in aged porcine oocytes. (**a**) The representative images of ROS in aged porcine oocytes. (**b**,**c**) The graphs showing quantified intracellular levels of ROS and GSH in aged porcine oocytes. (**d**) The graph showing relative mRNA expression of oxidative stress related *(CAT*, *GPX*, and *SOD1)* genes in aged porcine oocytes. ROS levels were quantified by relative fluorescence intensity in porcine oocytes, scale bar = 280 µm. Each experiment was independently repeated at least three times. Significant difference (* *p* < 0.05).

RV is known to protect cells against oxidative stress and to determine whether RV can protect porcine oocyte against oxidative stress. We first measured the levels of intracellular ROS, as shown in Figure 4a,b; the levels of ROS were significantly higher in the 24 h and 48 h untreated aged groups when compared with the control group, while the oocytes treated with 2 µmol/L RV (24 and 48 h) showed significantly higher fluorescence intensity levels as compared to the control group but significantly lower intensity as compared to the untreated aged groups. The glutathione (GSH) is an important intracellular antioxidant because it exerts powerful functions for protecting the cells from the oxidative stress-induced damage, and so the GSH and GSSG kits were used to detect the ROS levels. Surprisingly, our analysis for ROS levels using GSH and GSSG kit shows that the intracellular levels of GSH in the 24 h and 48 h untreated aged groups were significantly lower when compared with the control group (Figure 4c). The GSH content in the 24 h and 48 h groups treated with 2 µmol/L of RV was significantly higher than that in the 24 h and 48 h untreated aged groups. In addition, we also determined the mRNA expression of antioxidant and oxidative stress related genes *(CAT*, *GPX*, and *SOD1*) by qRT-PCR analysis. Our results (Figure 4d) showed that the mRNA transcript levels of *CAT* and *SOD1* groups’ oocytes treated with 2 µmol/L RV were significantly higher than those in the untreated aged groups; however, the expression of *CAT* and *SOD1* was lower as compared to the control group. Likewise, there was a significant increase in the treated groups of GPX, and no difference was found between the untreated aged *GPX* and control groups. 

### 2.5. RV Rescues the Mitochondrial Membrane Potential in Aged Porcine Oocytes

**Figure 5:**
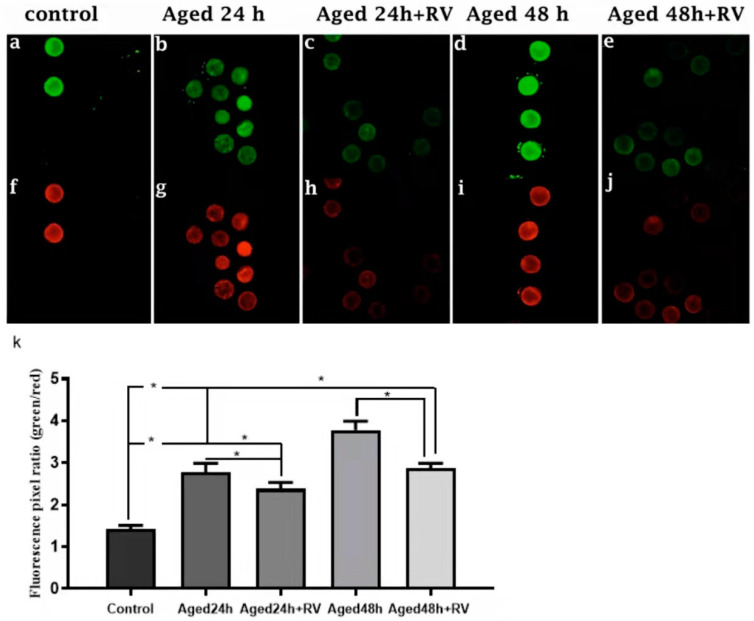
RV rescues the mitochondrial membrane potential in aged porcine oocytes. Representative Fluorescent images of JC-1-stained oocytes. Cultured in the absence or presence of 2 µmol/L RV. (**a**,**f**) Control; (**b**,**g**) aged 24 h; (**c**,**h**) aged 24 h + RV; (**d**,**i**) aged 48 h; (**e**,**j**) aged 48 h + RV. (**k**) Quantitative analysis of JC-1 red/green fluorescence intensity ratio in porcine oocytes. Membrane potential was calculated as the ratio of red fluorescence, which corresponds to activated mitochondria (J-aggregates), and to green fluorescence, which corresponds to less-activated mitochondria (J-monomers). Fluorescence emitted from each oocyte was analyzed using the ImageJ software. Significant difference (* *p* < 0.05).

It is well-known that oxidative stress is associated with mitochondrial membrane potential (MMP) changes and cell apoptosis. Therefore, we intended to determine the mitochondrial membrane potential state during porcine oocyte aging. To investigate the mitochondrial membrane potential, we analyzed the ratio of red/green fluorescence. As shown in Figure 5c,e,h,j,k, the oocytes treated with 2 µmol/L RV (24 h and 48 h) aged groups showed lower ratios than those in the untreated aged groups. Moreover, oocytes in control (Figure 5a,f,k) showed the lowest values. Based on our findings, this is concluded that RV has remarkable efficacy on keeping mitochondrial membrane potential in porcine oocyte aging. 

## 3. Discussion

One of the main aspects of ovarian aging is the decline in fertility over time, which is characterized by the decline in the quality and quantity of oocytes [34]. However, some evidence suggests that an imbalance between ROS and antioxidants causes a decline in oocyte quality, which is a critical factor in the success of ART and is linked to the aging of the ovaries. Furthermore, the RV as an antioxidant has been proved to alleviate oxidative stress in various cell types including oocytes. Moreover, RV has shown to prevent mitochondrial damage in cardiomyocytes through the upregulation of the deacetylation of apoptotic proteins. Studies have revealed that treatment of porcine oocytes with 2 µmol/L RV significantly reduced the levels of intracellular ROS while increased GSH contents during in vitro maturation [35,36]. In our study, we revealed that, under in vitro conditions, 2 μmol/L RV was able to delay postovulatory oocyte aging, owing to possible mechanisms mediated by reducing oxidative stress. RV could significantly increase the GSH content in 24 h and 48 h aged groups treated with 2 μmol/L of RV as compared with the control group (*p* < 0.05). The certain survival factors and antiapoptosis factors lead to oocyte maturation disorder or apoptosis by a decrease in mature-promoting factor (MPF) stability [37]. Furthermore, induced oxidative stress can adversely affect a variety of reproductive processes including sperm capacitation, ovulation, and corpus luteum production and can also trigger oocyte apoptosis. The accumulation of ROS has serious manifestation regarding the quality and aging of oocytes [38]. The uncontrolled and excessive production of free radicals may harm DNA, proteins, and lipids, which can severely compromise cell health and contribute to the disease development [39,40,41]. Our results showed that RV can significantly reduce the ROS level in aged oocytes (24 and 48 h), which is consistent with previous findings [42]. Similarly, RV (at concentrations ≥100 μM) has been shown to scavenge O2 directly in a nonenzymatic, cell-free system [43]. Findings in the present study indicate the potential of RV to delay oocyte aging by reducing ROS levels owing to its reported antioxidant [44], antiapoptosis [45], and antiaging [42] activities. Moreover, RV has also shown to reduce lipid peroxidation by eliminating free radicals and thus achieve the effect of protecting cells [46]. The main antioxidant enzymes are *SOD*, catalase (*CAT*), and glutathione peroxidase (*GPX*). Moreover, O_2_ is converted by *SOD* to H_2_O_2_, which is decomposed to water and oxygen by *CAT*, preventing the production of hydroxyl radicals. In addition, *GPX* transforms peroxides and hydroxyl radicals into nontoxic forms by oxidizing reduced glutathione (GSH) into glutathione disulfide and triggers reduction to GSH by glutathione reductase [47]. When Cu^+2^ or Fe^+2^ are available, H_2_O_2_ reacts with these ions to form unstable hydroxyl radicals. Previous studies have shown that RV can increase the expression of various antioxidant genes such as *CAT, SOD*, and *GPX* in cells [48,49]. When low-dose of RV was used to treat cardiomyocytes, the catalytic activity of *CAT* and *SOD* increased significantly with no effect on glutathione activity. Moreover, *SOD* can reduce intracellular superoxide levels and potentially resist against cell apoptosis, membrane permeability changes, and mitochondrial dysfunction [50]. Previous studies in our laboratory have demonstrated that RV can eliminate mitochondrial injury while delaying oocyte aging and improving the expression of sirtuin-1 (sirt1) and thus the quality of aged porcine oocytes [1]. Similarly, in the present study, RV treatment increased the expression of *GPX* gene in 24 h aged groups as compared to the control and untreated aged groups (*p* < 0.01). However, the expression of *CAT* and *SOD1* genes was lower than that of the control group (*p* < 0.05) but still higher than their untreated counterparts. Likewise, RV increases GSH content in primary keratinocytes and in epidermis of a reconstructed skin model as reported previously [51]. The antioxidant response of RV was further confirmed through enhanced activity of SOD with administration of 2-NP in a rat model conducted by Lodovici et al. [52]. Our findings revealed that RV can effectively mediate oxidative stress induced by the aging oocytes during in vitro culture through increasing the antioxidant gene expression. Progesterone causes elongation of the Mos poly (A) tail via cytoplasmic polyadenylation, and this polyadenylation increases the rate of Mos translation leading to the accumulation of Mos protein [53]. Mos protein is essentially required for the initiation of oocyte germinal vesicle breakdown [54]. In our study, we observed an increase in cumulus spread after treatment of oocytes with different concentrations of RV (0, 1, 2, and 4 µmol/L), which is consistent with previous findings, as mentioned earlier. Furthermore, PB1 extrusion rate with 2 μmol/L of RV group was also significantly higher than other groups, indicating the potential of RV to increase oocyte maturation rate in a dose-dependent manner. During this study, we found that oocytes treated with RV showed a significant decrease in perivitaline spaces during 24 h and 48 h of aging. However, differences were nonsignificant compared with the control group. A full expansion of cumulus cells is mandatory for the proper maturation of the oocyte. The beneficial effects of RV might depend upon its ability to improve oocyte quality. Therefore, it can be concluded that RV (2 μmol/L) can inhibit the increase of perivitaline space of oocytes aged for 24 h and 48 h, indicating its ability to alleviate the adverse effects of postovulatory oocyte aging by improving the quality, which is necessary for its development during fertilization. Apoptosis is well-known for exogenous (mediated by death receptors) and mitochondria-guided endogenous pathways. Both of these pathways participate in the activation of certain members of the Caspase family to trigger apoptosis. The proteins involved in the mitochondria-mediated endogenous pathway include the member of the Bcl-2 family, which comprises both antiapoptotic and proapoptotic proteins. The antiapoptotic proteins (Bcl-XL, Bcl-2, and Mcl-1) potentially inhibit the activation of the Caspase family and block the transduction of apoptotic signals, while proapoptotic proteins (Bcl XS, Bak, Bax, and Bad) promote and initiate an apoptotic response. Caspase 3 acts as a key effector in the process of apoptosis and directly hydrolyzes specific substrates. When there is apoptosis, Bax acts on the outer mitochondrial membrane of cell causing the release of mitochondrial cytochrome C (Cytc) that activates Caspase 3 and triggers Caspase cascade. The nucleated cytoskeleton recombines and degrades cytoskeletal structure [33]. However, Bcl 2 inhibits Cytc and Caspase, causing an antiapoptotic effect. The recruitment of Bax, which is knocked out, restricts the expression of Bax and leads to an increase in the number of ovarian oocytes [55]. Deacetylated Sirt1 and Sirt-3 inhibit the apoptotic pathway by affecting 1-methyl-4-phenyl-1, 2, 3, 6-tetrahydropyridine (MPTP) pores of the mitochondrial membrane [56]. Furthermore, downregulation of caspase 3 upregulates the expression of the antiapoptotic protein such as Bcl-2 that subsequently inhibits apoptosis. We observed lower expression of Bax and Caspase 3 in RV-treated aged oocytes in the present study. Moreover, relative mRNA expression of Bcl-2 was significantly lower in the RV-treated group than the control but was significantly higher as compared to untreated aged groups. Our findings suggested that RV can effectively inhibit mitochondrial apoptotic pathway through downregulation of the Bax and Caspase 3 while upregulating the expression of Bcl-2 in aged oocytes, eventually reducing the adverse effects of aging in porcine oocytes. Mitochondria are responsible for maintaining cellular metabolic functions, and their physiological efficiency can be assessed by examining the mitochondrial membrane potential state. In this regard, fluorescence probes such as JC-1 tend to accumulate in the mitochondrial matrix (by forming I-J-aggregates) and produce red excitation light when the mitochondrial membrane potential is maintained high. However, if mitochondrial membrane potential is maintained low, JC-1 cannot accumulate in the mitochondrial matrix and, hence, forms monomers and generates green excitation light. On the basis of these results is suggested a remarkable efficacy of RV on keeping mitochondrial membrane potential in porcine oocyte aging. During the present study, inclusion of RV in the oocyte culture medium maintained the mitochondrial membrane potential of aged oocytes in a state consistent with nonaged counterparts. Furthermore, RV significantly increased the expression of Bcl-2 in 24 and 48 h aged oocytes, which subsequently can modulate the mitochondrial apoptotic pathway by controlling the permeability of the outer mitochondrial membrane [57]. Consequently, the RV-regulated follicular development primarily through increased expression of mitochondrial-related Bcl-2 gene, which might have played its role in the maintenance of mitochondrial membrane potential in its normal position in 24 and 48 h aged oocytes. However, further in vivo studies are required to elucidate its potential mechanism of action.

## 4. Materials and Methods

### 4.1. Ethics Statement

The present study (short title: “Resveratrol hinders postovulatory aging by modulating oxidative stress in porcine oocytes”) was carried out in strict accordance with the recommendation of the National Ethical commission of (Nanjing, Jiangsu, China). All the experiments and procedures compiled with the guideline and were approved by the local ethical committee of the Nanjing Agricultural University (Nanjing, Jiangsu, China) with respect to animal experimentation and care of animal under study. 

### 4.2. Reagents 

Resveratrol (R5010, Sigma, purity ≥ 99%), Dulbecco’s PBS (DPBS), Hyaluronidase H-3506, DMSO D2650, paraformaldehyde 158127, poly vinyl alcohol (PVA) 046K0086, D-Mannitol M-9647, and Sodium pyruvate 100M12532V were purchased from Sigma–Aldrich (St. Louis, MO, USA), unless otherwise mentioned. 

### 4.3. Oocytes Collection and IVM 

The porcine ovaries were collected from prepubertal gilts at a local slaughterhouse of (Nanjing Yuan-run Group Co., Ltd., Nanjing, China) and transported to our laboratory at 37 °C in 0.9% NaCl (*w*/*v*) physiological saline within 2 h postcollection. Follicular fluid from superficial follicles of 3–6 mm in diameter was aspirated using a disposable syringe with an 18-guage needle, and the fluid was immediately transferred into conical tubes to allow COCs to settle down at the bottom for quick (pick up purpose) of COCs. After 10–12 min, the whole bottom sediment was placed down in petri dish. Follicular contents containing COCs that had more than 3 unexpanded cumulus cell layers with uniform cytoplasm were selected under a stereomicroscope (Olympus, Tokyo, Japan) and washed thrice in HEPES buffered Tyrode’s medium containing 0.05% (*w*/*v*) PVA (TLH–PVA). A group of approximately 50–70 of COCs was placed in each well in a 4-well plate (Nunclon, Roskilde, Denmark) containing 500 µL pre-equilibrated TCM199 medium (Gibco NY, USA) supplemented with 3.05 Mm D-glucose, 0.91 Mm sodium pyruvate, 0.57 Mm cysteine, 10 ng/mL epidermal growth factor, 10 IU/mL PMSG and hCG (Ningbo Hormonal Reagents Co., Ltd., Ningbo, Zhejiang, China), 75 µg/mL penicillin, 50 µg/mL streptomycin, 0.1% (*w*/*v*) polyvinyl alcohol, and 10% (*v*/*v*) porcine follicular fluid (pFF) [58], covered with 150 µL of mineral oil at 38.5 °C in an atmosphere of 5% CO_2_ in humidified air for 44 h.

### 4.4. RV Concentration and In Vitro Aging

RV was dissolved in 10 mmol/L of Dimethyl Sulfoxide (DMSO) as a stock solution and was stored at −20 °C before use. At the start of each culture, the stock solution was diluted with TCM-199 in vitro maturation medium to adjust a final concentration of 2 μmol/L for the RV treatments. For in vitro aging analysis, oocytes were cultured in IVM medium supplemented with or without 2 μmol/L RV (Control, Aged 24 h, Aged 24 h + RV, Aged 48 h, and Aged 48 h+ RV groups, respectively) for an additional 24 h and 48 h prolonged aging period at 38.5 °C supplemented with 5% CO_2_ in the humidified air for 44 h. The fresh oocytes without any prolonged culture were used as control group.

### 4.5. RNA Isolation and Quantitative Real-Time Polymerase Chain Reaction (qRT-PCR)

According to the time-based group differentiation and after maturation (44 h) of oocytes, the denuded oocytes were collected and washed thrice in DPBS solution and stored at −80 °C until the RNA was extracted. A total of 100 oocytes were used for total RNA extraction (in three replicates) from each group using the Trizol™ Reagent (Thermo Fisher scientific, Waltham, MA, USA). The extracted RNA was quantified using Nano-Drop and stored at −80 °C until further use. The first strand of cDNA was synthesized from 2 ug of total RNA with Primer Script™ RT Master Mix (Takara, Dalian, China) following the manufacturer’s described reaction protocol: 37 °C for 15 min, 85 °C for 5 s, and hold at 4 °C. The synthesized cDNA was subjected to real-time PCR using TB Green^®^ Premix Ex Taq™ (TaKaRa, Dalian, China). The forward and reverse primer sequences for real-time PCR are listed in Table 1. The reaction conditions were 30 s at 95 °C, followed by 40 cycles of 95 °C for 5 s and 60 °C for 30 s. Ultimately, they were quantified at 95 °C for 15 s, 60 °C for 1 min, and 95 °C for 15 s. At least three replications were performed for each reaction and data were analyzed using the 2^−^^△△CT^ method [58]. 

### 4.6. Measurement of Reactive Oxygen Species (ROS) Intensity

To measure the level of intracellular ROS, DCFH-DA (2, 7-Dichlorodi-hydrofluorescein diacetate) and 10 µM working solution (with TCM199 medium), after diluting together, were equilibrated in the incubator at 37 °C for 30 min subsequently, and the oocytes were incubated in the DCFH-DA working solution at 37 °C for 1 h under total darkness. After incubation, oocytes were washed three times in PBS, and the fluorescence signals were detected and imaged using confocal microscope (Zeiss LSM 700META, Oberkochen, Germany) that was fluorescence intensity of ROS (Excitation wavelength: 450–490 nm and Emission wavelength: 515–565 nm). The relative fluorescence intensity was measured with ImageJ 1.5 software (Bethesda, Maryland, USA). Total numbers of 25–30 oocytes (in three replicates) were used for ROS measurement, respectively.

### 4.7. Determination of Intracellular GSH Contents

The contents of total glutathione (T-GSH) were examined through an enzymatic method by using a GSH/GSSG assay kit (Beyotime, Shanghai, China) according to the manufacturer’s instructions. A total of 50 oocytes from each group were placed into a small conical tube containing 30 µL of protein scavenger M solution supplied with the kit. Afterward, tube contents were vortexed thoroughly for 5 min, then the mixture was frozen at liquid nitrogen for 2 min and thawed in a water bath at 37 °C repeatedly for 3 times. Subsequently, mixture was centrifuged at 10,000 rpm for 10 min at 4°C and placed on ice for 5 min using a 96-well plate. The samples or standard in the sequence were added and mixed accordingly. Immediately, absorbance was observed at 405 nm with a microplate reader, for 25 min, with a reading recorded for every 5 min. A standard curve was developed for the determination of the GSH content of each sample. The GSH concentration was calculated by dividing the total concentration of each sample by the total number of oocytes present in the sample (pmol/oocyte). 

### 4.8. Mitochondrial Membrane Potential Assay

The mitochondrial membrane potential (MMP, ∆φm) of the aged and fresh oocytes was evaluated using mitochondrial membrane potential assay kit JC-1 (Beyotime, Shanghai, China). The oocytes were exposed to 10 μL JC-1 in 100 μL working solution at 38.5 °C in 5% CO_2_ for 20 min under total darkness. To remove surface fluorescence, oocytes were washed three times in PBS and then mounted on glass slides using D-PBS for microscopy. Laser excitation was set at 488 nm for green and 525 nm for red fluorescence, respectively. The fluorescence microscope (Zeiss LSM 700 META, Oberkochen, Germany) with the same scan settings for each sample was used to measure the fluorescence intensity of each oocyte. ImageJ 1.5 software (Bethesda, Maryland, USA) was used to analyze the normal fluorescence pixel intensities of each oocyte. The ratio of green to red fluorescence pixels was used to analyze mitochondrial membrane potential. 

### 4.9. Statistical Analysis

Each treatment group had a minimum of 3 replicates, and the images of oocytes stained in the same dye were captured with the same scan settings. The average value of fluorescence intensity in each group of oocytes was analyzed after deduction of the background fluorescence through ImageJ software (National Institutes of Health, Bethesda, MD, USA). The obtained data were analyzed by the Statistical Package for Social Sciences (SPSS) software (version18.0) by using one-way analysis of variance (ANOVA). The treatment means were compared by the least significant difference (LSD) test at 1% and 5% probability levels. The *p*-value of <0.05 was considered a significant difference, while *p* < 0.01 was considered as highly significant, and *p* < 0.001 was considered as extremely significant. 

## 5. Conclusions

Findings of the present study revealed that RV can effectively alleviate the adverse effects of oocyte aging by increasing the expression of antioxidant enzymes while decreasing the ROS level. Additionally, the RV treatment resisted against the decline in mitochondrial membrane potential in aged oocytes. Moreover, RV showed potent antiapoptotic effects by potentially upregulating the expression of Bcl-2 while downregulating the Bax and Caspase 3 transcript levels. Collectively, our findings lead to the evidence that RV may be one of the important constituents in improving the oocyte quality by delaying the antiaging effects through its antioxidant properties on porcine oocytes.

## Figures and Tables

**Table 1:** Primer sequences of the target genes used for RT-qPCR.

Target Gene	Forward and Reverse Sequence	Product Size (bp)	Accession Number
GAPDH	F: 5′-GTCGGTTGTGGATCTGACCT-3′ R: 5′-TTGACGAAGTGGTCGTTGAG-3′	207	NM_001206359
Caspase-3	F: 5′-CGTGCTTCTAAGCCATGGTG-3′ R: 5′-GTCCCACTGTCCGTCTCAAT-3′	186	NM_214131
Bcl-2	F:5′-AGGGCATTCAGTGACCTGAC-3′ R: 5′-CGATCCGACTCACCAATACC-3′	193	NM_214285
Bax	F:5′-TGCCTCAGGATGCATCTACC-3′ R: 5′-AAGTAGAAAAGCGCGACCAC-3′	199	XM_003127290
CAT	F-5′-AACTGTCCCTTCCGTGCTA-3′ R-5′-CCTGGGTGACATTATCTTCG-3′	195	XM_021081498
GPX	F:GAGCCCTTCAACCTGTCCTC R:GTCGGACGTACTTCAGGCAA	210	NC_010455.5
SOD1	F-5′-ACCTGGGCAATGTGACTG-3′ R-5′-TCCAGCATTTCCCGTCT-3	131	NM_001190422

## Data Availability

The data that support the findings of this study are available on a reasonable request from the corresponding author.

## Sample Availability

Samples of the compounds are not available from the authors.
